# Novel coronavirus (2019-nCoV) early-stage importation risk to Europe, January 2020

**DOI:** 10.2807/1560-7917.ES.2020.25.4.2000057

**Published:** 2020-01-30

**Authors:** Giulia Pullano, Francesco Pinotti, Eugenio Valdano, Pierre-Yves Boëlle, Chiara Poletto, Vittoria Colizza

**Affiliations:** 1 INSERM, Sorbonne Université, Institut Pierre Louis d’Epidémiologie et de Santé Publique, IPLESP, Paris, France; 2 Center for Biomedical Modeling, The Semel Institute for Neuroscience and Human Behavior, David Geffen School of Medicine, University of California Los Angeles, Los Angeles, United States

**Keywords:** 2019-nCoV, importation risk, Europe, travel ban

## Abstract

As at 27 January 2020, 42 novel coronavirus (2019-nCoV) cases were confirmed outside China. We estimate the risk of case importation to Europe from affected areas in China via air travel. We consider travel restrictions in place, three reported cases in France, one in Germany. Estimated risk in Europe remains high. The United Kingdom, Germany and France are at highest risk. Importation from Beijing and Shanghai would lead to higher and widespread risk for Europe.

Starting December 2019, cases of pneumonia of unknown aetiology were reported in the city of Wuhan, in the province of Hubei in China [1]. The infective pathogen was later identified as a novel coronavirus, called 2019-nCoV [2]. As at 26 January 2020, a total of 1,988 confirmed cases have been reported from China [3]. The main affected area is in the province of Hubei, but as at 27 January 2020, confirmed cases have also been reported in 32 other provinces [4].

Forty-one travel-related cases were confirmed as at 27 January 2020, all coming from China. Twenty-seven cases were imported to Asia, six to North America, five to Oceania, and three to Europe [3,5-7]. Thirty of them were exported from Wuhan. In Europe, all three cases were imported to France. They were confirmed on 24 January 2020, with travel dates on 18 January 2020 (2 cases) and 22 January 2020 (1 case). One case was confirmed in Germany on 27 January 2020 with no history of travel to China but contact with a Chinese guest visiting their company [8]. In an effort to contain the spread of the virus, Chinese authorities enforced a travel ban in the province of Hubei starting on 23 January 2020 (3 a.m. Central European Time). This includes a complete ban on international flights [9].

Here we estimate the risk of importation of 2019-nCoV cases to Europe from infected areas in China by air travel. We compare the risk prior to the travel ban in Hubei province, with the risk updated to the outbreak situation of 27 January 2020, accounting for three cases imported to France and one case confirmed in Germany.

## Modelling risk of importation

For this study, Europe is defined according to the Wikipedia contemporary geographical definition but with exclusion of transcontinental countries (Azerbaijan, Georgia, Kazakhstan, Russia and Turkey) [10]. The risk of importation to Europe is estimated as the probability that at least one case is imported from China to Europe. It is based on estimates from the platform EpiRisk [11] and accounts for origin-destination air travel flows of January 2019 from the Official Airline Guide (OAG) database of the GLEAM Project [11-13]. Details of the computation are provided in the Supplementary Material.

To estimate the risk in Europe prior to the travel ban in the Hubei province, we consider Wuhan as the only seed of the international spread [3,5-7]. We then provide a colour-coded map of Europe to report for each country the probability that a case imported to the continent arrives there, when coming from Wuhan only. For sensitivity, we tested whether the risk changes considering air travel flows of the month of February 2019.

**Figure 1:**
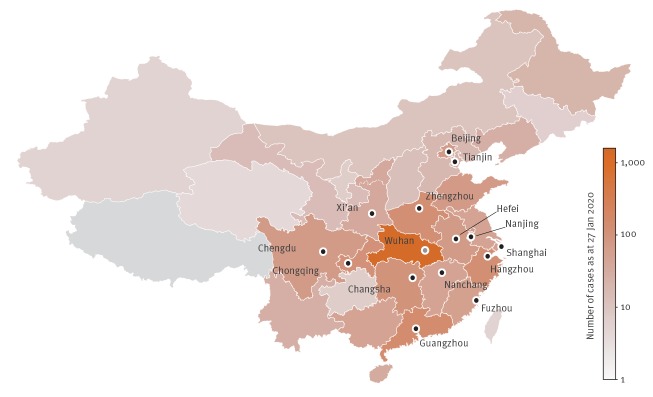
Map of Chinese provinces colour coded according to the number of cases of 2019-nCoV [4] as at 27 January 2020

To estimate the risk in Europe following the travel ban, we consider as possible seeds of case exportation out of China the cities that are highly connected to Wuhan based on de-identified and aggregated domestic population movement data (2013–2015) derived from Baidu Location-Based Services [14]. These cities are depicted in Figure 1. They were also found to be highly correlated with those reporting a high number of cases in the corresponding provinces [14].

2019-nCoV: Novel coronavirus.

The 14 cities selected for the multi-source seeding [14] are shown with black dots: Beijing (in the province of Beijing), Changsha (Hunan), Chengdu (Sichuan), Chongqing (Chongqing), Fuzhou (Fujian), Guangzhou (Guangdong), Hangzhou (Zhejiang), Hefei (Anhui), Nanchang (Jiangxi), Nanjing (Jiangsu), Shanghai (Shanghai), Tianjin (Tianjin), Xi’an (Shaanxi), Zhengzhou (Henan). Wuhan (grey dot) is subject to the current travel ban.

To account for the current situation, including the three cases in France and one in Germany, we estimate the risk of importation to Europe except France and Germany as the probability that Europe (France and Germany excluded) imports at least one travel-related case from China, conditioned to the observation of three cases imported to France and one case in Germany. Details of the computation are provided in the Supplementary Material. We estimate the risk for a varying number of exported cases from China, cumulative in time, to account for likely detection delays or under-detection of travel-related cases. As before, we then provide a colour-coded map of Europe to report for each country the probability that a case imported to the continent arrives there, when coming from cities depicted in Figure 1, except Wuhan. For sensitivity, we also tested whether the risk changes due to the additional inclusion of Wuhan in the multi-source seeding.

## Estimated importation risk from Wuhan before the travel ban in Hubei province

**Figure 2:**
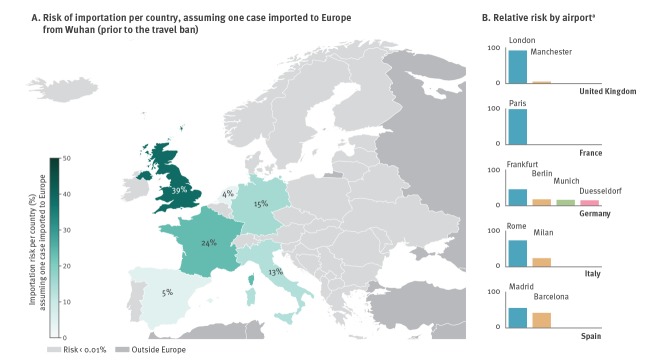
(A) Country-specific risk of importation assuming one case imported to Europe from Wuhan before the travel ban, and (B) relative risk by airport^a^, January 2020

^a^ When a city is served by several airports, these airports are considered as one entity.

The exportation of 30 cases from Wuhan before the travel ban, as reported so far, was estimated to put Europe at 61% risk of importing at least one case. The risk was localised in Western European countries, with the highest risk estimated for the United Kingdom (UK; 39%), followed by France (24%), and Germany (15%) (Figure 2). In some countries, importations are likely to occur at multiple airports (e.g. Germany, Italy, Spain), whereas in others the risk is mostly concentrated in airports serving the capital city (e.g. London in the UK, and Paris in France).

No change was estimated to occur when considering travel flow data from the month of February (data not shown).

## Estimated importation risk from considered areas of China following the travel ban in Hubei province

**Figure 3:**
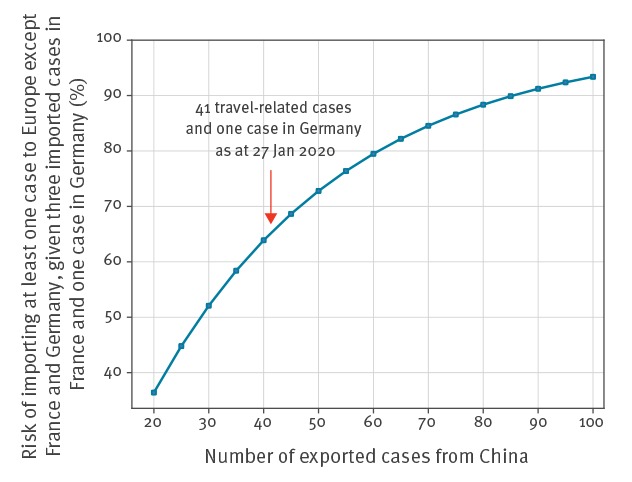
Risk, as a function of the cumulative number of exported cases from China, of importing at least one case to Europe except France and Germany, given three imported cases reported in France and one case confirmed in Germany, January 2020

The probability that at least one case is imported to Europe except France and Germany, given the three imported cases reported in France and one case confirmed in Germany, is high (Figure 3). It is estimated to be more than 64% for the number of travel-related exportations from China reported so far (41 travel-related and one confirmed case in Germany). The probability becomes larger than 80% if 60 cases are exported from China.

**Figure 4:**
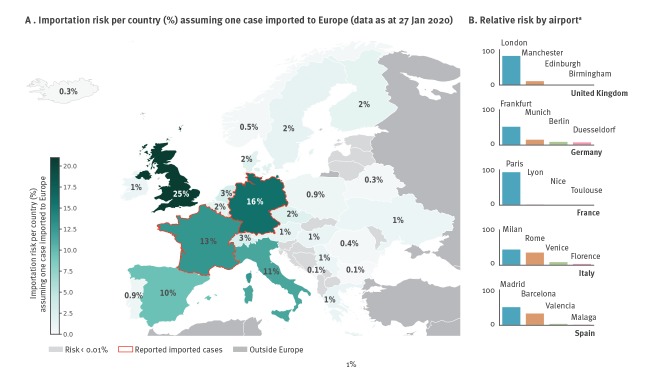
(A) Country-specific risk of importation assuming one case imported to Europe from the multi-source seeding of Figure 1 and (B) relative risk by airport^a^, January 2020

^a^ When a city is served by several airports, these airports are considered as one entity. For each country, only the four most important cities in terms of agglomeration of airports and passenger traffic are represented.

In the event that one travel-related case is imported to Europe, the risk of importation is highest in the UK (25%) (Figure 4). Germany and France, which already have confirmed cases, rank second and third with a probability of 16% and 13% to receive another case, respectively. Italy (11%) and Spain (9.5%) rank as fourth and fifth in terms of risk. The risk is in general higher in more populated countries (Supplementary Figure S1). Also Eastern Europe and Northern Europe would be at risk of importing cases.

Updated to data as at 27 January 2020.

In the UK and France, the airports serving the capital cities continue to contribute the largest likelihood of importing cases (London contributes to 83% of the risk, Paris contributes to 94% of the risk, respectively).

The estimates account for the travel ban imposed in the province of Hubei. Including travel flows from Wuhan, to account for cases who may have flown before the travel ban and are not yet detected, does not alter the estimations (data not shown).

## Discussion and conclusions

France reported on 24 January 2020 the importation of three 2019-nCoV confirmed cases from China, and Germany confirmed its first case on 27 January 2020 with no history of travel to China. They are still the first and only imported cases confirmed in Europe, at the time of writing. We estimate that the risk of importation of at least one case to Europe except France and Germany is high. It is larger than 80% if 60 travel-related cases are exported from China. The three countries at highest risk are the UK, Germany, and France (confirming estimates reported by other studies [12,14,15]), with the latter two countries already reporting cases. Delays are expected from date of importation to date of identification that may bias observations at the time of writing. All three cases imported to France were confirmed on 24 January 2020, with two travelling on 18 January 2020 (6 days delay) and one on 22 January 2020 (2 days delay).

The risk pattern of 2019-nCoV importation estimated for Europe varies considerably depending on the geographical extent of the affected areas in China. In particular, a larger area acting as seed of exportation that includes Shanghai and Beijing (two cities with larger number of travellers to more widespread areas in Europe) would likely result in a higher and more widespread risk for Europe.

Our results are based on available data and estimates of the affected provinces in China and account for origin-destination travel fluxes from these provinces, as well as the travel ban enforced in the Hubei province. However, estimates are sensitive to different health-seeking behaviours that infected travellers may have, and to the active surveillance practices put in place in European countries. We did not provide estimates of the expected number of imported cases per country, as this depends on the number of travel-related exported cases from China, a variable that is still hard to assess at this early stage.

Risk maps will need to be rapidly updated as the outbreak situation evolves. 

## Supplementary Data

102000 Supplementary Material
