# Supplementary material for: Novel coronavirus (2019-nCoV) early-stage importation risk to Europe, January 2020
Source: Euro Surveill. 2020 Jan 30;25(4):2000057. doi: 10.2807/1560-7917.ES.2020.25.4.2000057 (PMC7001240; doi:10.2807/1560-7917.ES.2020.25.4.2000057)
Supplement: Supplementary Material [file 2000057_COLIZZA_Supplementary_Material.pdf]

SUPPLEMENTARY MATERIAL

Novel coronavirus (2019-nCoV) early-stage importation risk to Europe, January 2020

Giulia Pullano[1], Francesco Pinotti[1], Eugenio Valdano[2], Pierre-Yves Boëlle[1], Chiara Poletto[1], Vittoria Colizza[1]

1. INSERM, Sorbonne Université, Institut Pierre Louis d'Epidémiologie et de Santé Publique IPLESP Paris, France.
2. Center for Biomedical Modeling, The Semel Institute for Neuroscience and Human Behavior, David Geffen School of Medicine, University of California Los Angeles, Los Angeles, USA.

This text provides computation details of the methods illustrated in the main paper, and a plot of additional results.

This supplementary material is hosted by Eurosurveillance as supporting information alongside the article "Novel coronavirus (2019-nCoV) early-stage importation risk to Europe, January 20", on behalf of the authors, who remain responsible for the accuracy and appropriateness of the content. The same standards for ethics, copyright, attributions and permissions as for the article apply. Supplements are not edited by Eurosurveillance and the journal is not responsible for the maintenance of any links or email addresses provided therein.

Computation of the probability of importing at least 1 case in Europe

Considering a given infected area in China acting as seed of exportation, p_{EU} is the probability that an individual travels from that area to Europe computed on the travel flows. If x cases are exported, the risk of importation of at least 1 case to Europe is computed as $1-(1-p_{EU})^x$.

Computation of the probability of importing 1 case in Europe except France, conditioned to observing 3 imported cases in France

Let us define:
n : number of cases exported from China;
m : number of countries in Europe with detected cases;
$\vec{x}$: m-dimensional vector encoding the number of cases in each European country with detected cases;
$c=\sum_{j=1}^{m} x_j$: number of detected cases imported to Europe;
$\vec{p}$: m-dimensional vector encoding the importation probabilities in each European country with detected cases;
$g=\sum_{j=1}^{m} p_j$: probability of importing to any of the m countries;
q : probability of importing to Europe except the m countries;
y : cases potentially imported to Europe except the m countries.

We need $P(y|\vec{x})$. We decompose it as follows, and compute separately the numerator and the denominator:

$$P(y|\vec{x}) = \frac{P(y, \vec{x})}{P(\vec{x})}$$

Both numerator and denominator come from multinomial distributions. The distribution for the denominator is $(m+1)$-dimensional: probability of importing to one of the m countries, and probability to import somewhere else. The distribution for the numerator is $(m+2)$-dimensional: probability of importing to one of the m countries, to import somewhere to Europe except the m countries, and probability to import somewhere else.
Putting these together, we get

$$P(y|\vec{x}) = \binom{n-c}{y}\left(1 - \frac{q}{1-g}\right)^{n-c} \left(\frac{q}{1-g-q}\right)^{y}.$$

Setting $y = 0$, we can compute the probability of having at least one case as

$$P(y > 0|\vec{x}) = 1 - \left(1 - \frac{q}{1-g}\right)^{n-c}.$$

Country-specific importation risk vs. country population size

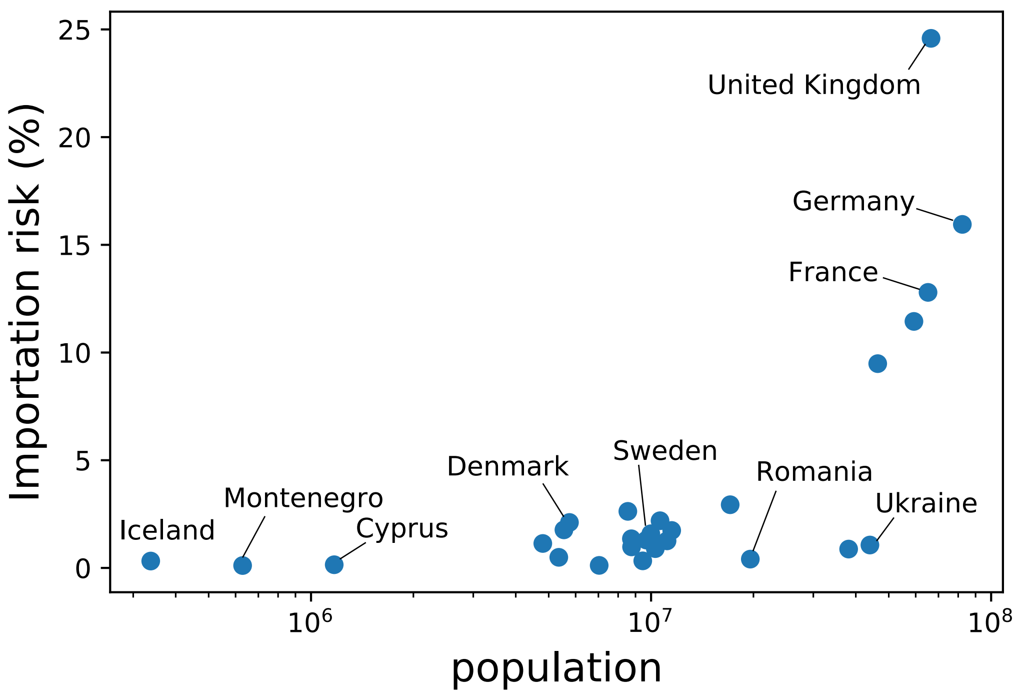

FIGURE S1: The estimated country-specific importation risks are shown as a function of the country population sizes.
